# Are Ghanaian women meeting the WHO recommended maternal healthcare (MCH) utilisation? Evidence from a national survey

**DOI:** 10.1186/s12884-021-03643-6

**Published:** 2021-02-23

**Authors:** Edward Kwabena Ameyaw, Kwamena Sekyi Dickson, Kenneth Setorwu Adde

**Affiliations:** 1 School of Public Health, Faculty of Health, University of Technology Sydney, Sydney, NSW Australia; 2 Department of Population and Health, College of Humanities and Legal Studies, University of Cape Coast, Cape Coast, Ghana

**Keywords:** Maternal healthcare, Antenatal, Postnatal, Skilled birth, Ghana, Public health

## Abstract

**Background:**

To achieve the Sustainable Development Goal target 3.1, the World Health Organisation recommends that all pregnant women receive antenatal care (ANC) from skilled providers, utilise the services of a skilled birth attendant at birth and receive their first postnatal care (PNC) within the first 24 h after birth. In this paper, we examined the maternal characteristics that determine utilisation of skilled ANC, skilled birth attendance (SBA), and PNC within the first 24 h after delivery in Ghana.

**Methods:**

We used data from the 2014 Ghana Demographic and Health Survey. Women aged 15-49 with birth history not exceeding five before the survey were included in the study. A total of 2839 women were included. Binary logistic regression was employed at a 95% level of significance to determine the association between maternal factors and maternal healthcare (MCH) utilisation. Bivariate and multivariate regression was subsequently used to assess the drivers.

**Results:**

High proportion of women had ANC (93.2%) with skilled providers compared to the proportion that had SBA (76.9%) and PNC within the first 24 h after delivery (25.8%). Only 21.2% utilised all three components of MCH. Women who were covered by national health insurance scheme (NHIS) had a higher likelihood (AOR = 1.31, CI = 1.04 – 1.64) of utilising all three components of MCH as compared to those who were not covered by NHIS. Women with poorer wealth status (AOR = 0.72, CI = 0.53 – 0.97) and those living with partners (AOR = 0.65, CI = 0.49 – 0.86) were less likely to utilise all three MCH components compared to women with poorest wealth status and the married respectively.

**Conclusion:**

The realisation that poorer women, those unsubscribed to NHIS and women living with partners have a lower likelihood of utilising the WHO recommended MCH strongly suggest that it is crucial for the Ministry of Health and the Ghana Health Service to take pragmatic steps to increase education about the importance of having ANC with a skilled provider, SBA, and benefits of having the first 24 h recommended PNC.

## Background

According to the World Health Organisation (WHO) classification, Ghana has a high maternal mortality ratio (MMR) [1]. This is expected given that Ghana was not able to achieve the MDG target of 190 MMR per 100,000 live births in 2015 [2]. A recent estimate indicates 310 MMR and this places Ghana among countries with high MMR globally [1, 3]. However, Ghana seeks to achieve the Sustainable Development Goal (SDG) target 3.1, thus 70 MMR by 2030 [4]. This can only be achieved when women can utilise the full continuum of maternal healthcare (MCH) irrespective of location, socio-economic, or demographic traits. The WHO strongly recommends that all pregnant women should have antenatal care (ANC) with a skilled provider, utilise the service of a skilled birth attendant (SBA) at birth and obtain first postnatal care (PNC) within the first 24 h after birth [5, 6].

A greater section of women in Ghana is unable to meet these recommendations due to several setbacks. It has been documented that poor women [7, 8] the uneducated or least educated [8–10] and rural residents [10, 11] least utilise these recommended life-saving services. Similarly, socio-cultural beliefs and convictions sometimes dissuade women from accessing these services [12, 13]. The socio-cultural issues sometimes manifest in the limited decision-making capacity of women at the household level and the thought that women who give birth in health facilities are weak [14].

To reverse the narrative, the Government of Ghana and the Ministry of Health have instituted some edge-cutting interventions within the past two decades. The National Health Insurance Scheme (NHIS), for instance, was effected in 2005 to cushion the poor [15]. The free MCH policy was subsequently added to the insurance scheme in 2008 to ensure that women who are subscribed to the insurance scheme enjoy all MCH services without any out-of-pocket payment [16, 17]. Likewise, the Community-based Health Planning Services (CHPS) was introduced in 2008 to the fulfilment of the Universal Health Coverage (UHC) target and to reach persons in rural and remote locations with essential health care including maternal health services [18]. In spite of all these, the current MMR is disturbing and Ghana is unlikely to achieve the SDG target 3.1 by 2030.

Admittedly, a plethora of evidence exists on MCH utilisation in Ghana [10, 19–21]. A few of these studies have investigated all three cardinal MCH utilisation indicators (antenatal, childbirth, and postnatal) [8, 10]. However, none of the previous studies investigated these three MCH dimensions recommended by the WHO; (1) having ANC with a skilled provider, (2) birth with a skilled birth attendant, and (3) having the first PNC within the first 24 h after childbirth. This study sets to fill this gap in knowledge by investigating the maternal characteristics that determine the utilisation of the service of a skilled ANC provider, skilled birth attendant, and attaining one’s initial PNC within the first 24 h after childbirth. The outcome of this study could enlighten the government and all stakeholders to plan and design effective and fit for purpose MCH interventions and expedite the nation’s prospects of achieving the SDG target 3.1.

## Material and methods

### Data source

The Ghana Demographic and Health Survey (GDHS) used a standard DHS model questionnaire developed by the Measure DHS programme [22]. This study used the most recent DHS data and a descriptive cross-sectional study design. The DHS are national surveys carried out every 5 years in low- and middle-income countries globally [23]. The surveys concentrate on maternal and child health, physical activity, sexually transmitted infections, fertility, health insurance, tobacco use, and alcohol consumption. They mainly provide data to monitor the demographic and health profiles of the respective countries [23]. For the purpose of the study, women with birth history who had given birth up to 5 years before the survey were included. A sample of 2839 women with complete data required for our analysis participatedble in this study. Permission to use the data set was given by the MEASURE DHS following the assessment of our concept note. The DHS program permitted us to use the dataset after evaluation of our concept note. The datasets are freely available to the public at www.measuredhs.com.

### Description of variables

Outcomes of interest were skilled providers of antenatal care (ANC), skilled birth attendance, and postnatal care utilisation within 24 h. Skilled provider of ANC and skilled birth attendance were derived from the question “did you see anyone for antenatal care for this pregnancy? If YES: whom did you see?” and “who assisted with the delivery?” respectively. Responses were categorized under Health Personnel and Other Person. Health personnel included doctor, nurse, nurse/midwife, an auxiliary midwife; Other persons also consisted of the traditional birth attendant (TBA), traditional health volunteer, community/village health volunteer, neighbours/ friends/relatives, other. For the purpose of the study, skilled birth attendance referred to births assisted by a doctor, nurse, auxiliary midwife, nurse/midwife [21, 24–26]. Postnatal utilisation within 24 h was derived from the question “How many hours, days, or weeks after the birth of (NAME) did the first check take place?” The response was recoded as within 24 h (coded as 1) and after 24 h (coded as 0).

Twelve explanatory variables were used for the study. These include age, place of residence, level of education, covered by health insurance, frequency of listening to radio, frequency of watching television, wealth status, antenatal care visits, marital status, getting medical help for self: getting permission to go, getting medical help for self: getting money needed for treatment, getting medical help for self: distance to health facility.

### Statistical analysis

The study employed both inferential and descriptive analysis. The inferential analysis was a multivariate binary logistic analysis of the predictors of the outcome variables. Four models were employed. Model 1 was based on the background characteristics and skilled ANC provider. Model 2 and 3 were constructed on background characteristics and skilled birth attendance and postnatal care respectively. Model 4 is a combination of all the outcome variables and background characteristics.

Descriptive analysis was computed by having a bivariate analysis of the 12 explanatory variables and the outcome variables. All analyses were done using STATA version 14 and all results were weighted. The presence of multicollinearity between the independent variables was checked before fitting the models. The variance inflation factor (VIF) test revealed the absence of high multicollinearity between the variables with mean VIF = 3.68 among explanatory variables. Categories with the highest frequencies were used as the reference groups throughout the analysis.

### Ethics approval

This study benefited from publicly available data from DHS. Permission to access the data was granted by the Measure DHS Program. Pre-approval was obtained from all participants prior to the survey. The DHS Program adheres to ethical standards to protect respondents’ privacy. Inner-City Fund (ICF) International also ensures that the surveys are in line with the ethical requirements of the US Department of Health and Human Services. No additional ethical approval is required because the data is secondary and publicly available. Details of the ethical standards are available on http://goo.gl/ny8T6X.

## Results

### Descriptive analysis

Nine in ten women utilised the service of a skilled ANC provider during antenatal care visits. Seventy-six percent of women were assisted by a skilled birth attendant during delivery. Nearly 3 in ten women received postnatal care within 24 h as prescribed by the WHO (Table 1). Only 2 in 10 women utilised all the three components of MCH.

**Table 1:** Background characteristics and MCH Utilisation

**Variable**	**Frequency** ***N* = 2839**	**Utilised Skilled ANC provider** **%**	**Utilised Skilled Birth Attendance** **%**	**Utilised Post-natal Care within 24 h** **%**	**Utilised all three** **%**
***Age***					
15 – 19	67	94.3	73.8	28.5	26.7
20 – 24	387	92.1	71.6	28.7	21.0
25 – 29	699	93.7	78.1	19.9	16.6
30 – 34	735	94.4	77.8	32.1	27.1
35 – 39	584	93.2	81.4	25.8	21.6
40 – 44	274	93.0	75.9	21.4	18.3
45 – 49	93	83.4	59.6	20.3	13.4
***Place of residence***					
Urban	1352	97.3	91.7	26.1	24.3
Rural	1487	89.4	63.4	25.6	18.4
***Level of education***					
No education	794	88.1	57.5	25.2	19.2
Primary	531	91.2	71.9	29.6	21.7
Secondary	1369	96.3	87.7	23.9	20.9
Higher	145	99.0	100.0	34.3	34.3
***Covered by health insurance***					
No	875	90.8	70.2	23.6	17.6
Yes	1965	94.2	79.9	26.9	22.8
***Frequency of listening to radio***					
Not at all	426	90.2	64.1	23.4	17.0
Less than once a week	960	91.2	73.9	23.3	18.6
At least once a week	1453	95.4	82.6	28.2	24.2
***Frequency of watching television***					
Not at all	758	87.4	59.3	23.0	14.6
Less than once a week	772	93.3	78.6	25.0	20.4
At least once a week	1309	96.5	86.1	28.0	25.6
***Wealth status***					
Poorest	637	85.6	51.5	25.6	17.0
Poorer	535	89.9	61.2	20.8	14.2
Middle	466	93.1	80.3	23.3	18.1
Richer	566	97.8	95.0	26.7	25.5
Richest	635	99.4	96.9	31.4	30.0
***Antenatal care visits***					
Less than 4	278	71.0	36.3	21.9	8.7
4 or more	2561	95.6	81.3	26.3	22.6
***Marital status***					
Married	1995	93.9	77.9	27.2	22.2
Living with partner	618	91.1	74.6	20.2	17.2
Widowed	51	86.6	48.5	37.0	22.7
Divorced	43	95.9	91.7	31.3	29.2
Separated	132	93.9	79.4	26.3	22.1
***Getting medical help for self: getting permission to go***					
Big problem	150	89.6	71.6	10.2	6.7
Not a big problem	22,689	93.4	77.2	26.7	22.1
***Getting medical help for self: getting money needed for treatment***					
Big problem	1144	89.3	67.9	22.3	16.2
Not a big problem	1695	95.8	83.0	28.3	24.6
***Getting medical help for self: distance to health facility***					
Big problem	708	89.9	61.4	21.4	12.4
Not a big problem	2131	94.3	82.1	27.3	24.2
Total	2839	93.2	76.9	25.8	21.2

Twenty-seven percent of women age 30 – 34 years utilised all the three components of MCH. Women from urban areas (24.3%), with higher education (34.3%), covered with health insurance (22.8%), with richest wealth status (30.0%) and had 4 or more antenatal care visit (22.6%) utilised all the three components of MCH (see Table 1). Women who did not have a big problem getting permission [i.e. significant difficulty in seeking permission from home] (22.1%), getting money needed for treatment (24.6), and distance to health facility (24.2%) utilised all the three component of MCH (see Table 1).

### Multivariate analysis

Results from all the models are shown in Table 2. This section highlights the model that combines all the three components of MCH utilisation. Our study showed that level of education, wealth status, marital status, antenatal care utilisation, coverage of health insurance, getting medical help for self: getting permission to go and distance to health facility had significant association with MCH utilisation. Women who were not covered by NHIS had a lesser likelihood (AOR = 0.76, CI = 0.61 – 0.96) of utilising all three components of MCH as compared to those who were covered by NHIS. Also, women with poorer wealth status (AOR = 0.72, CI = 0.53 – 0.97) and those living with partners (AOR = 0.65, CI = 0.49 – 0.86) were less likely to utilise all three components of MCH compared to women with poorest wealth status and the married respectively (see Table 2).

**Table 2:** Logistic regression on MCH utilisation

**Variables**	**Utilised Skilled ANC provider**	**Utilised Skilled Birth Attendant**	**Utilised Post-natal Care with 24 h**	**Utilised all three**
	**AOR (CI)**	**AOR (CI)**	**AOR (CI)**	**AOR (CI)**
***Age***				
15 – 19	2.50 (0.81-7.71)	1.76 (0.91-3.39)	0.52 (0.26-1.03)	0.77 (0.38-1.58)
20 – 24	1.05 (0.64-1.71)	1.21 (0.88-1.68)	0.94 (0.71-1.24)	0.95 (0.70-1.30)
25 – 29	1.10 (0.71-1.71)	1.13 (0.85-1.49)	0.72** (0.57-0.92)	0.74* (0.57-0.96)
30 – 34	Ref	Ref	Ref	Ref
35 – 39	0.79 (0.51-1.22)	1.38* (1.02-1.87)	0.71** (0.55-0.92)	0.74* (0.56-0.97)
40 – 44	0.98 (0.57-1.68)	1.45* (1.01-2.08)	0.82 (0.59-1.11)	0.94 (0.67-1.32)
45 – 49	0.55 (0.30-1.02)	1.01 (0.63-1.62)	0.67 (0.42-1.09)	0.72 (0.42-1.24)
***Place of residence***				
Urban	Ref	Ref	Ref	Ref
Rural	0.58** (0.38-0.90)	0.45*** (0.34-0.60)	1.08 (0.86-1.37)	0.94 (0.73-1.22)
***Level of education***				
No education	0.94 (0.62-1.41)	0.49*** (0.38-0.60)	1.29* (1.01-1.65)	1.35* (1.03-1.77)
Primary	0.91 (0.59-1.39)	0.73** (0.55-0.97)	1.54** (1.19-1.97)	1.31 (0.99-1.73)
Secondary	Ref	Ref	Ref	Ref
Higher	1.57 (0.20-12.05)	1	1.31 (0.86-1.99)	1.40 (0.91-2.13)
***Covered by health insurance***				
No	0.94 (0.68-1.28)	0.63*** (0.51-0.78)	0.84 (0.69-1.03)	0.76** (0.61-0.96)
Yes	Ref	Ref	Ref	Ref
***Frequency of listening to radio***				
Not at all	0.92 (0.61-1.36)	0.63** (0.48-0.82)	0.91 (0.70-1.16)	0.92 (0.69-1.21)
Less than once a week	0.69 (0.49-1.16)	0.70** (0.56-0.89)	0.83 (0.67-1.03)	0.81 (0.64-1.02)
At least once a week	Ref	Ref	Ref	Ref
***Frequency of watching television***				
Not at all	0.78 (0.51-1.20)	1.05 (0.79-1.36)	0.94 (0.73-1.21)	0.81 (0.61-1.078)
Less than once a week	0.69* (0.49-0.98)	0.86 (0.65-1.15)	0.86 (0.67-1.11)	0.74* (0.56-0.97)
At least once a week	Ref	Ref	Ref	Ref
***Wealth status***				
Poorest	Ref	Ref	Ref	Ref
Poorer	1.38 (0.93-2.00)	1.11 (0.86-1.43)	0.76* (0.58-0.99)	0.72* (0.53-0.97)
Middle	1.40 (0.85-2.31)	1.67** (1.20-2.32)	0.92 (0.67-1.25)	0.97 (0.69-1.36)
Richer	2.44* (1.16-5.13)	5.05*** (3.03-8.43)	0.98 (0.69-1.39)	1.17 (0.80-1.71)
Richest	9.39** (2.06-42.78)	4.92*** (2.48-9.77)	1.12 (0.75-1.68)	1.31 (0.85-2.01)
***Antenatal care visits***				
Less than 4	0.22*** (0.16-0.30)	0.24*** (0.18-0.32)	0.87 (0.64-1.16)	0.51** (0.34-0.75)
4 or more	Ref	Ref	Ref	Ref
***Marital status***				
Married	Ref	Ref	Ref	Ref
Living with partner	0.69* (0.48-0.99)	0.76* (0.59-0.99)	0.59*** (0.46-0.76)	0.65** (0.49-0.86)
Widowed	0.95 (0.43-2.12)	0.66 (0.36-1.21)	1.55 (0.89-2.67)	1.50 (0.82-2.75)
Divorced	1.43 (0.41-5.0)	3.82** (1.48-9.90)	0.98 (0.48-2.00)	1.23 (0.57-2.65)
Separated	0.70 (0.33-1.46)	0.97 (0.57-1.65)	0.77 (0.47-1.25)	0.70 (0.40-1.22)
***Getting medical help for self: getting permission to go***				
Big problem	1.09 (0.64-1.87)	1.25 (0.84-1.85)	0.38*** (0.24-0.61)	0.38** (0.22-0.67)
Not a big problem	Ref	Ref	Ref	Ref
***Getting medical help for self: getting money needed for treatment***				
Big problem	0.54*** (0.39-0.76)	0.90 (0.72-1.12)	0.87 (0.71-1.07)	0.94 (0.75-1.18)
Not a big problem	Ref	Ref	Ref	Ref
***Getting medical help for self: distance to health facility***				
Big problem	1.17 (0.84-1.62)	0.66*** (0.53-0.83)	0.99 (0.79-1.23)	0.74** (0.57-0.95)
Not a big problem	Ref	Ref	Ref	Ref

*Ref* Reference Category, *AOR* Adjusted Odds Ratio, *CI* Confidence Interval

*p < 0.05 **p < 0.01 **p < 0.001

Our study revealed that women who made less than four antenatal care visits had a lesser odd (AOR = 0.51, CI = 0.34 – 0.75) of utilising all three MCH services compared to those who made four or more ANC visits. Similarly, women who had a big problem getting permission to seek medical help for themselves had a lesser likelihood (AOR = 0.38, CI = 0.22 – 0.67) of utilising all three MCH services compared to those who stated they did not have a big problem getting permission to seek health care (see Table 2). Women who stated that distance was a big problem in getting medical care had a lesser odds (AOR = 0.74, CI = 0.57 – 0.95) of utilising all three MCH services as compared to those who asserted that distance to a health facility was not a big problem in getting medical care (see Table 2).

## Discussion

Three key components of MCH to reduce MMR are SBA [24, 27], having ANC with a skilled provider [28, 29] and PNC [30]. However, the MMR in Ghana remains high and this necessitated the study to unveil the proportion of women in Ghana having SBA, obtaining ANC with skilled providers, and having PNC within the first 24 h after delivery as well as the associated drivers for policy interventions.

The study showed that the majority of women in Ghana utilised skilled ANC and SBA, however, the majority of the women did not receive PNC within 24 h as prescribed by WHO [5, 6]. The high proportion of women using ANC services in this study corroborates the findings of Dickson et al. [25] who also observed that about 9 out of 10 women receive ANC services in Ghana from a skilled provider. Although Ghana records a relatively high proportion of women using SBA, the same cannot be said for other SSA countries such as Niger (32.6%), Mali (39.9%), and Sierra Leone (45.2%) [24].

Regarding the utilisation of PNC services within 24 h after delivery, Wudineh et al. [30] similarly observed a low utilisation of PNC services within the first 24 h in Ethiopia (30.5%). The high ANC and SBA in Ghana can be attributed to the interventions instituted by the Ministry of Health and the Government of Ghana over the past two decades. For instance, the NHIS and the free MCH policies have been instituted to enable the poor and less privileged in the country to be able to access MCH [16, 17]. The low utilisation of PNC within 24 h could be as a result of healthcare providers not counselling women on the importance of PNC within the first 24 h. The study also observed that only a few proportions of women utilised all three components of MCH. This could largely be attributed to the low utilisation of PNC within 24 h.

The study revealed that level of education, wealth status, marital status, antenatal care utilisation, coverage of health insurance, getting medical help for self: getting permission to go and distance to health facility have significant relationship with MCH utilisation. Ayele, Melku, and Belda [31] similarly observed that place of residence, mother’s education, travel time, joint decision on the place of delivery, birth preparedness and complication readiness status, frequency of ANC visit, knowledge of obstetric danger signs and knowledge of the presence of maternity waiting homes significantly influence the utilisation of SBA. Bhowmik, Biswas, and Woldegiogis [32] further indicate that place of residence, age at first birth, wealth index, working status, participation in household decisions, and partner and respondent’s education significantly predict SBA utilisation.

We found that women with poorer wealth status were less likely to utilise all three maternal health services. The results of another study conducted in Ghana showed that women within the richest wealth status were more likely to utilise ANC service [21]. A possible explanation for this is that women within the richest wealth status can afford possible MCH costs outside the remit of the NHIS (e.g. transportation cost and cost of further laboratory examinations) as compared to the poor. It was also observed that women living with partners were less likely to utilise all three MCH services as compared to those who were married. This affirms another study in Ghana which observed that cohabiting women were less likely to utilise MCH [33].

We also found that women who had less than four ANC visits were less likely to utilise all three components of MCH as compared to their counterparts who made four or more ANC visits. This corroborates previous findings that ANC attendance has positive correlation with MCH utilisation [33, 34]. A possible explanation is that women who do not derive satisfaction from ANC visits are less likely to have more ANC visits, which then translates into limited utilisation of other MCH services.

The study showed that women who had a big problem in getting permission to seek medical help for themselves had a lesser likelihood of MCH utilisation. The current findings affirm that of previous studies which also observed that a woman’s autonomy to visit a health facility serves as an enabler to utilising MCH services and vice versa [35, 36]. In Ethiopia, Bayu et al. [37] observed that women who do not have the support of their partners do not obtain permission to seek healthcare and are less likely to have SBA compared with those who obtain such support. A possible deduction is that the patriarchal system in Ghana, coupled with other cultural and traditional beliefs serve as barriers to the utilisation of MCH services in Ghana and thereby reducing the likelihood of MCH utilisation among women who find it difficult to get permission [15].

Previous studies in Burkina Faso [38], Ghana [39], Nigeria [40], and Cambodia [41] observed that distance was a major barrier for women in accessing MCH services. Similarly, this study observed that women who stated that distance was a big problem in getting medical care had a lesser odds of MCH utilisation compared to those who indicated that distance to a health facility was not a big problem in getting medical care. This could be attributed to the fact that the cost of travelling, travel time, and means of transportation are barriers to seeking MCH [41].

## Strengths and limitations

This study has some noteworthy strengths. It was based on a large nationally representative sample. This makes it possible for our findings to be generalised to all women in the reproductive age group in Ghana. However, a cross-sectional study design was followed in executing the study, and hence, the deduction of causal inference is not possible. Since the outcome was not directly observed but self-reported, there is the possibility of recall bias especially with respect to the timing of the first PNC.

## Conclusions

The findings strongly suggest that it is crucial for the Ministry of Health and the Ghana Health Service to take pragmatic steps to increase education about the importance of having ANC with a skilled provider, SBA, and having the first 24 h recommended PNC. The findings also demonstrate that level of education, wealth status, marital status, antenatal care utilisation, coverage of health insurance, getting medical help for self: getting permission to go and distance to health facility have a significant association with MCH utilisation. This indicates that tailored educational and pro-MCH interventions must be streamlined to acknowledge the nuances conditioned by these factors.

## Data Availability

All analysed data are freely available to the public through https://dhsprogram.com/data/dataset/Ghana_Standard-DHS_2014.cfm?flag=0. Permission to access the data was granted by the Measure DHS Program.

## Abbreviations

ANC: Antenatal care
AOR: Adjusted odds ratio
CI: Confidence interval
CHPS: Community-based Health Planning Services
GDHS: Ghana Demographic and Health Survey
ICF: Inner-City Fund
MCH: Maternal and child health
MMR: Maternal mortality ratio
NHIS: National Health Insurance Scheme
PNC: Postnatal care
SBA: Skilled birth attendance
SDG: Sustainable Development Goal
TBA: Traditional birth attendant
UHC: Universal Health Coverage
VIF: Variance inflation factor
WHO: World Health Organisation
